# Inflammatory Microenvironment of Skin Wounds

**DOI:** 10.3389/fimmu.2022.789274

**Published:** 2022-03-01

**Authors:** Zhen Wang, Fang Qi, Han Luo, Guangchao Xu, Dali Wang

**Affiliations:** ^1^ Department of Plastic Surgery and Burns, Affiliated Hospital of Zunyi Medical University, Zunyi, China; ^2^ The Collaborative Innovation Center of Tissue Damage Repair and Regeneration Medicine of Zunyi Medical University, Zunyi, China

**Keywords:** wound healing, inflammatory microenvironment, inflammatory cells, non-inflammatory cells, extracellular matrix

## Abstract

Wound healing is a dynamic and highly regulated process that can be separated into three overlapping and interdependent phases: inflammation, proliferation, and remodelling. This review focuses on the inflammation stage, as it is the key stage of wound healing and plays a vital role in the local immune response and determines the progression of wound healing. Inflammatory cells, the main effector cells of the inflammatory response, have been widely studied, but little attention has been paid to the immunomodulatory effects of wound healing in non-inflammatory cells and the extracellular matrix. In this review, we attempt to deepen our understanding of the wound-healing microenvironment in the inflammatory stage by focusing on the interactions between cells and the extracellular matrix, as well as their role in regulating the immune response during the inflammatory stage. We hope our findings will provide new ideas for promoting tissue regeneration through immune regulation.

## Introduction

Wound healing involves the cooperation between several different cell types and the involvement of various growth factors, cytokines, and the extracellular matrix (ECM), which together contribute to three overlapping phases: inflammation, proliferation, and remodelling (1). Inflammation initiates the process of wound healing, which is critical to protect the body from pathogens and to remove necrotic tissue. However, excessive inflammation can lead to the persistence of chronic non-healing wounds, preventing the wound from proceeding to the remodelling stage, thereby delayed healing (2). To strictly control the balance between inflammatory activation and inhibition, and to prevent excessive or abnormal inflammatory reactions, it is necessary to comprehensively understand the inflammatory microenvironment of skin wounds.

**Figure 1:**
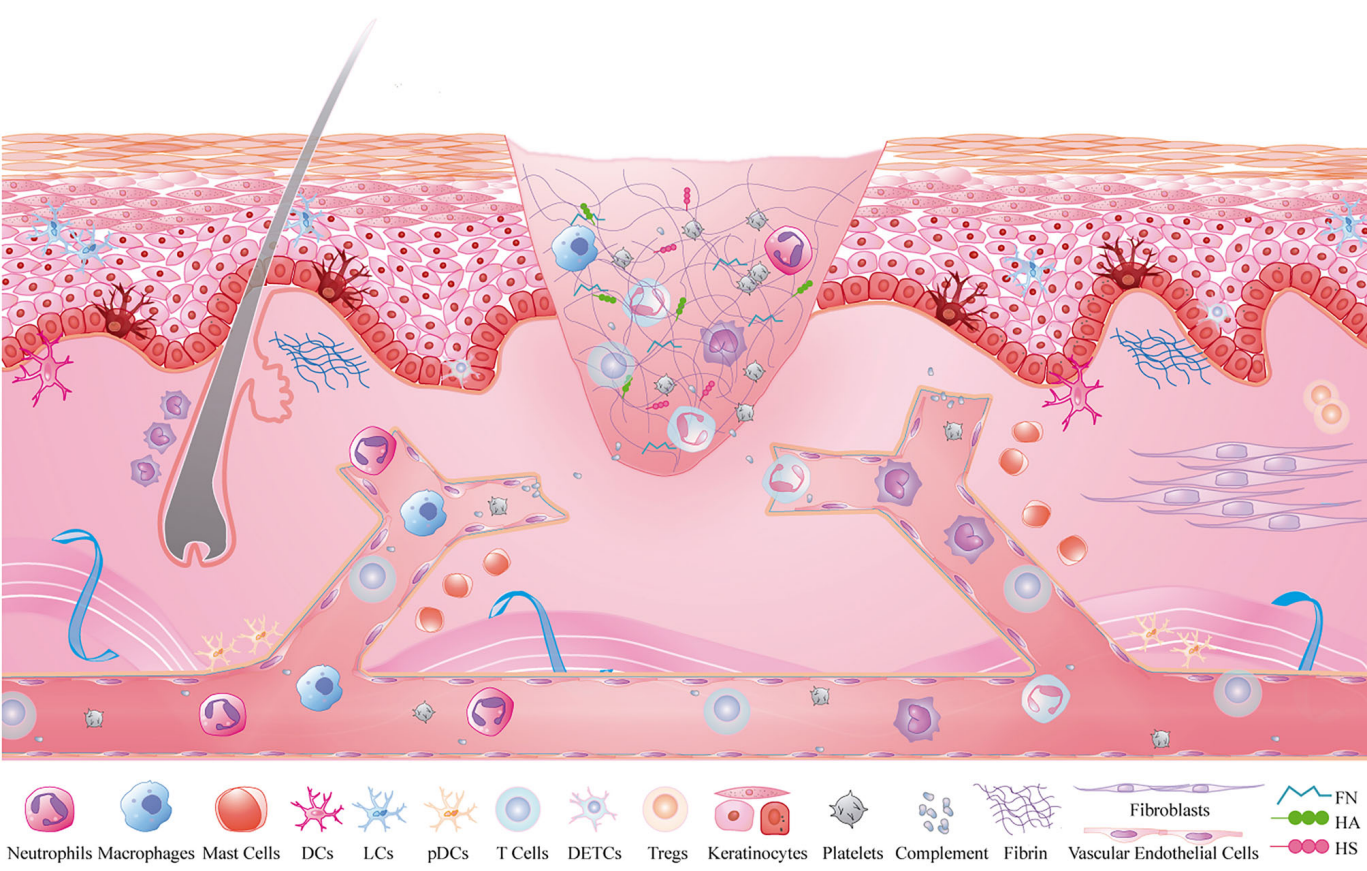
Inflammatory microenvironment of skin wounds. Inflammatory cells: neutrophils, macrophages, mast cells, T cells, DCs, platelets and complement; non-inflammatory cells: fibroblasts, keratinocytes and vascular endothelial cells; extracellular matrix: fibrin, FN, HA and HS.

Crosstalk activation between the platelet cascade reaction and the complement system initiates the wound repair process. As the first circulating cells are recruited to the inflammatory microenvironment, neutrophils play a role in the resistance to bacterial invasion (3). Subsequently, monocytes are recruited within 48-96 h after injury and transformed into tissue-activated macrophages (4). At the same time, the adaptive immune system, including mast cells, dendritic cells (DCs), and T lymphocytes, is activated against self and foreign antigens (1, 5). Inflammatory cells play an important role in the inflammatory stage during wound healing, but the function of non-inflammatory cells and the ECM cannot be ignored. Some subsets of non-inflammatory cells, such as keratinocytes, fibroblasts, and vascular endothelial cells, also play a role in regulating immunity (6). In addition to providing support and protection, the ECM is intimately associated with the adhesion and migration of immune cells (7). Therefore, inflammatory cells, non-inflammatory cells, and the ECM constitute the immune microenvironment of a wound, and their interactions are key to the regulation of wound healing (**Figure 1**).

In this review, we provide an up-to-date and detailed overview of the contribution of inflammatory cells, non-inflammatory cells, and the ECM to the wound microenvironment. We will focus on the non-inflammatory cells and the ECM in our review, which are two topics that have not received as much attention from a clinical perspective. Finally, we discuss how cell-cell and cell-ECM interactions regulate immune response in the wound microenvironment.

## Role of Inflammatory Cells in the Inflammatory Microenvironment

The inflammatory cascade is activated after haemostasis and coagulation. Neutrophils, macrophages, and lymphocytes respond to injury signals and are recruited from circulation to the wound microenvironment in a specific spatiotemporal order (8). Additionally, there are resident immune cells, including mast cells, DCs, and T lymphocytes (9). Together, they constitute the inflammatory cells of the wound inflammatory microenvironment and play an immunomodulatory role after skin injury.

### Neutrophils

Neutrophils are the first circulating immune cells that are recruited to the wound microenvironment. They can remove bacteria and necrotic tissue by releasing protease-containing particles or by degrading microorganisms in phagocytic cups. In addition, the inflammatory cascade depends on the hydrogen peroxide gradient formed by the neutrophil-releasing enzyme complex nicotinamide adenine dinucleotide phosphate oxidase (NADPH) and reactive oxygen species (ROS) (10). ROS participate in redox signal transduction to promote cell migration, proliferation, and renewal. Activated neutrophils also form prominent extracellular structures called neutrophil extracellular traps (NETs) (11). These structures extend to the extracellular microenvironment by nuclear budding or chromatin release to capture and eliminate pathogens. However, their high cytotoxic potential also means that their activation and migration need to be strictly regulated to prevent excessive inflammation from damaging tissue. Neutrophils not only inhibit healing by releasing soluble mediators and excessive ROS but they also secrete particles with pro-inflammatory microRNAs (miR-23a and miR-155) that can cause tissue damage (12). Wong et al. showed the deletion of key NETosis enzymes, or the inhibition of the “Nod-like receptor protein(NLRP3) inflammasome-NETs” inflammatory loop, can improve angiogenesis and accelerate wound healing (13). The neutropenia model can accelerate the speed of wound epithelial closure without changing the overall quality of the wound healing process. Therefore, timely removal of neutrophils from the inflammatory microenvironment is of great significance for wound healing.

**Figure 2:**
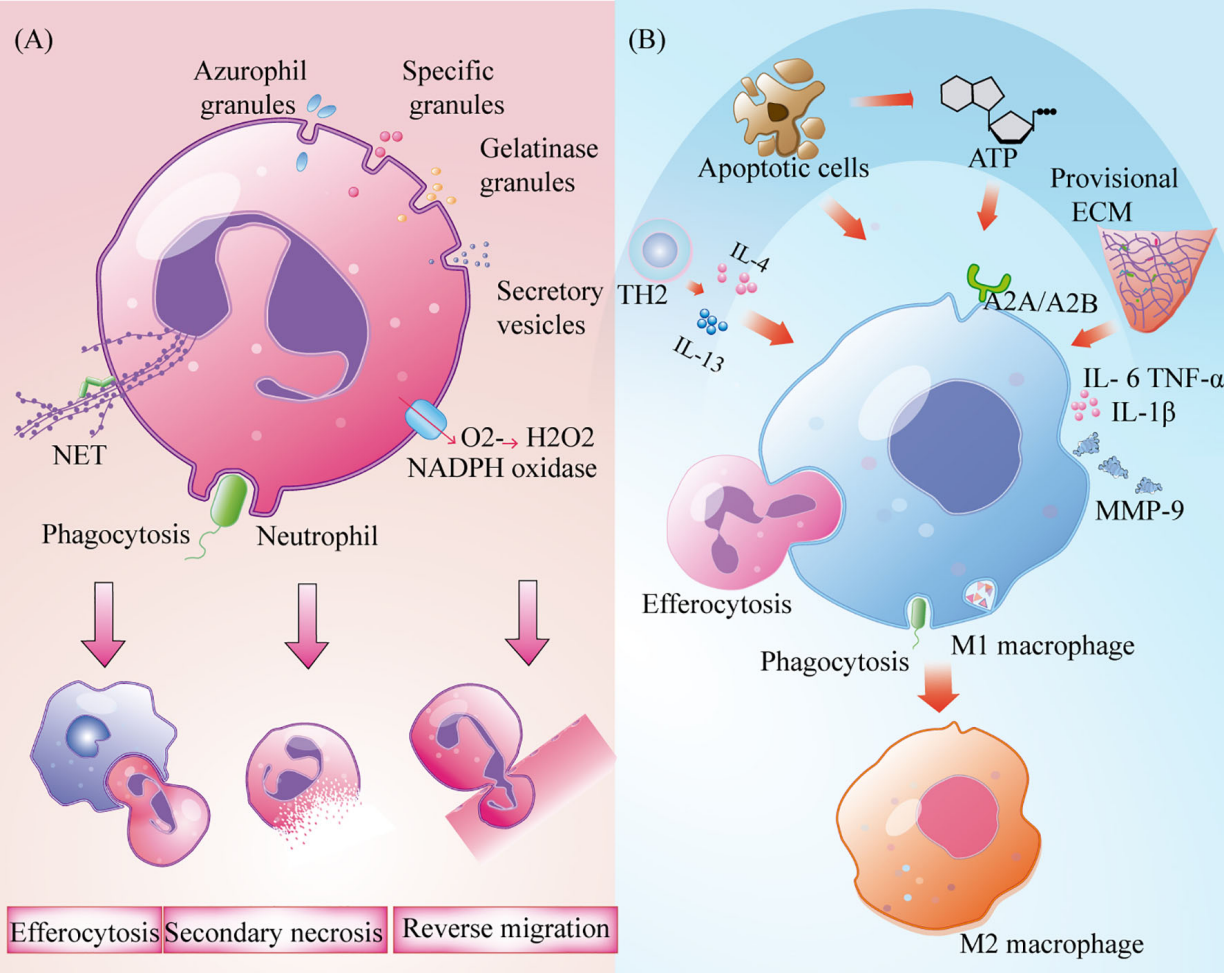
Role of the neutrophils and macrophages in wound inflammation. **(A)** Neutrophils produce extracellular trapping nets of neutrophils, trigger phagocytosis, produce oxidative bursts and degranulation to remove bacteria and necrotic tissue. It,s cleared by burial, reverse metastasis of neutrophils and secondary necrosis of neutrophils; **(B)** Macrophages removal cell fragments, pathogens and apoptotic cells, and produce a large number of pro-inflammatory cytokines to recruit immune cells. Polarization to M2 type under the influence of TH2 cytokines, apoptotic cells, nucleotides and temporary extracellular bases.

Neutrophils undergo apoptosis or necrosis after the removal of bacteria and necrotic tissue and are engulfed by macrophages through efferocytosis. A subset of neutrophils leave the injured site and return to the circulatory system by neutrophil reverse migration (14). However, neutrophils that are not cleared by these two processes develop secondary necrosis, resulting in the release of pro-inflammatory factors and cytotoxic molecules that result in tissue damage (**Figure 2A**). Clearing neutrophils from damaged areas is one of the principles of hyperbaric oxygen therapy that can enhance the healing ability of diabetic ulcer and chronic wound patients (15).

### Macrophages

After neutrophils are established, circulating monocytes quickly enter the tissue in response to injury signals and transform into macrophages after exposure to the local inflammatory microenvironment (1, 8). At the same time, a small number of resident macrophages near skin capillaries quickly recognise injury signals by expressing purine-producing receptors and promote the recruitment of inflammatory cells to the injured site (16). Although macrophages are involved in all stages of repair, their phenotypes and functions are regulated by the surrounding environment (17). Based on their different roles in wound repair, macrophages can be roughly assigned to three types: inflammatory macrophages, decomposing macrophages, and tissue remodelling macrophages (17). M1 macrophages that express high levels of the cell surface markers lymphocyte antigen 6 complex(Ly6c) and CC-chemokine Receptor 2(CCR2) are the main activated phenotypes in the inflammatory microenvironment. They act as phagocytes, as they remove cell fragments and pathogens, and clean wounds (18). When they phagocytise many apoptotic neutrophils, they can induce the progression from an inflammatory to a proliferative microenvironment (4, 19). These macrophages also produce and secrete high-levels of pro-inflammatory factors such as tumour necrosis factor-α (TNF-α), interleukin 1β (IL-1β), and cyclooxygenase-2, as well as other inflammatory cells that are recruited to participate in pathogen clearance (20). Lucas et al. and Goren et al. administered diphtheria toxin to lysM-Cre/DTR mice and observed a rapid decrease in the number of macrophages in injured skin, which resulted in serious damage to the wound shape and delayed healing (21, 22). In a prospective randomized trial, treatment of deep second-degree burns with recombinant human granulocyte macrophage colony-stimulating factor increased tissue macrophage count, enhanced immune response to local wounds, and ultimately accelerated wound healing (23). Although M1 macrophages have strong antibacterial ability, their persistence at the wound site may lead to MMP9 secretion and tissue damage (20). Therefore, the timely polarization of M1 macrophages is very important to promote the process of wound healing. In diabetic mice, wound healing was promoted through enhancement of the polarization from M1 to M2 (24). T helper-2 (Th2) cytokines and apoptotic cells that are present in the inflammatory microenvironment can promote the polarisation of M1 macrophages. This is accomplished under the synergistic action of nucleotides and the temporary ECM (25, 26) (**Figure 2B**). The world’s first treatment of diabetic foot ulcers using macrophage regulation (R&D code: ON101) has successfully completed a phase III clinical trial, demonstrating that targeting macrophage phenotypes is an effective method for the clinical treatment of chronic wounds (27).

### Mast Cells

Mast cells are immune cells that originate from the bone marrow and are located around blood vessels in the dermis, peripheral nerves, sebaceous glands and sweat glands (28, 29). They are the key effectors in mediating allergic reactions, and their role in host resistance to bacterial invasion has also been widely recognized. Tissue injury activates mast cells and releases prefabricated or *de novo* mediators into the inflammatory microenvironment, such as histamine, serotonin, and secretory granules (e.g., chymotrypsin, elastase, and trypsin) (28–30). Among them, histamine promotes skin wound healing by upregulating the expression of basic fibroblast growth factor (bFGF) to recruit macrophages and promote angiogenesis during the period of inflammation (31). The combination of trypsin and PAR2 on vascular endothelial cells causes telangiectasia and mediates neutrophil infiltration to the injured site (32). Inflammatory mediators, such as TNF-α, MMP-2, and IL-8, have also been shown to be effective in recruiting neutrophils and activating macrophages in the inflammatory phase (5). Studies on mast cell deficiency in mice have shown that it is closely related to impaired neutrophil recruitment (33). In some cases, however, too many mast cells can hinder wound healing. A study on mast cells in the skin of patients with type 2 diabetes showed that high expression of IL-3R mast cell clusters may be associated with chronic skin inflammation (34). McS-01, an indole-carboxyamide-type mast cell stabilizer developed by Tellechea et al., was shown to polarize macrophage phenotypes to accelerate wound healing in diabetic mice (35). However, many studies have shown that mast cells play a role in the proliferation stage of wound healing, especially in granulation tissue formation, fibroblast proliferation, and angiogenesis (36, 37). Scarless healing was transformed into scar formation by injecting mast cell lysates into mouse embryonic wounds (38). Notably, different microenvironmental stimuli can lead to differences in mast cell subtypes (39).

### DCs

Different subsets of DCs in the skin are recruited after tissue injury, including epidermal Langerhans cells (LCs), dermal DCs, and plasmacytoid DCs (pDCs). LCs are anchored to adjacent keratinocytes by the adhesion molecule E-cadherin (40). LCs maintains their count through self-renewal, and inflammatory stimulation enhances LC migration and local proliferation (41, 42). The formation of long and intricate dendritic structures between keratinocytes facilitates their rapid response to tissue damage (43). After skin injury, LCs migrate to lymph nodes under the stimulation of pro-inflammatory cytokines, IL-1β and granulocyte macrophage colony stimulating factor, and present injury or pathogenic antigens in the wound microenvironment to CD4T cells to induce an immune response (42). A previous study has shown that the greater the number of LCs in diabetic foot ulcers, the better is the healing effect, suggesting that human LCs play a beneficial role in the inflammatory wound-healing microenvironment (44). Clinical zinc supplementation in patients with bedsore can induce a high proportion of LC expression in wound marginal epidermis and significantly accelerate the healing process (45). However, different results were obtained when langerin(+) cells and CD11c+ cells were depleted in Lang-DTR and CD11C-DTR mouse models, respectively. The former promoted healing while the latter induced the opposite (46). The different results may be due to the use of different mouse LC depletion models. Therefore, while targeting LCs holds great potential for clinical wound healing, gaps in its understanding of the skin wound healing process need to be addressed. There are several other subtypes of dermal DCs. CD141+ DCs, which stimulate CD8+ T cell immunity by secreting IL-12 and thereby promote Th-1 differentiation (47, 48). CD1C+ DCs present CD4+ T cell antigens (47). In addition, after a skin injury, pDCs enter the inflammatory microenvironment and express Toll-like receptor (TLR)-7 and TLR-9, which recognise viral nucleic acids and induce an early inflammatory response (47, 49). A lack of pDCs in the wound healing microenvironment impairs acute inflammatory responses and delays wound healing (49). With the development of single-cell sequencing, the origin of DCs and their development in skin will be further clarified.

### T Lymphocytes

There are many different types of T lymphocytes in the epidermis and dermis. Dendritic epidermal T cells (DETCs) are the main type of T cells in the epidermis and form a cross-network with LCs by extending dendrites to the basal layer (50). DETCs are involved in the process of skin wound healing with the participation of mTOR, aromatic receptors, and STAT signaling pathway. DETCs become round after tissue injury and release CCL-3, CCL-4, CCL-5 and lymphocyte chemokines to regulate the migration of inflammatory cells (51, 52). In the presence of DETCs defects, wound closure in injured mice is delayed (52). Tissue injury around dermal hair follicles induces the accumulation of a large number of activated CD4+ helper T cells (Th) in the skin. Five major Th subgroups are known: Th1, Th2, Th17, Treg (T regulatory cells), and Tfh (follicular T helper cells). All Th subgroups show differences in cytokine release after skin injury, such as decreased IFNγ secretion by Th1 and increased IL-10 and IL-17 release by Th2 and Th17 (53). Th1 and Th2 can activate M1 and M2 macrophages, respectively, and Th17 can continuously recruit neutrophils; however, the direct impact of these cell types on wound healing is still unknown (54). Tregs are currently becoming the target of research into the relationship between Th and skin trauma. The aggregation of Tregs limits or eliminates damage in the early stages of trauma-related inflammation. When Tregs were depleted during the inflammation phase in the skin of injured mice, significantly higher levels of pro-inflammatory macrophages were observed and wound closure kinetics were significantly delayed (55). Independent of their immunomodulatory function, Tregs have also been found to have a regenerative effect that promotes cell differentiation (56). In addition, constant natural killer T cells promote wound healing by preventing the prolonged inflammation that is mediated by neutrophils and have been shown to accelerate wound healing in mice (43). Clearly, there are many permanent and circulating lymphocytes that are recruited to the wound healing microenvironment, but their different functions need to be further clarified.

### Platelets

**Figure 3:**
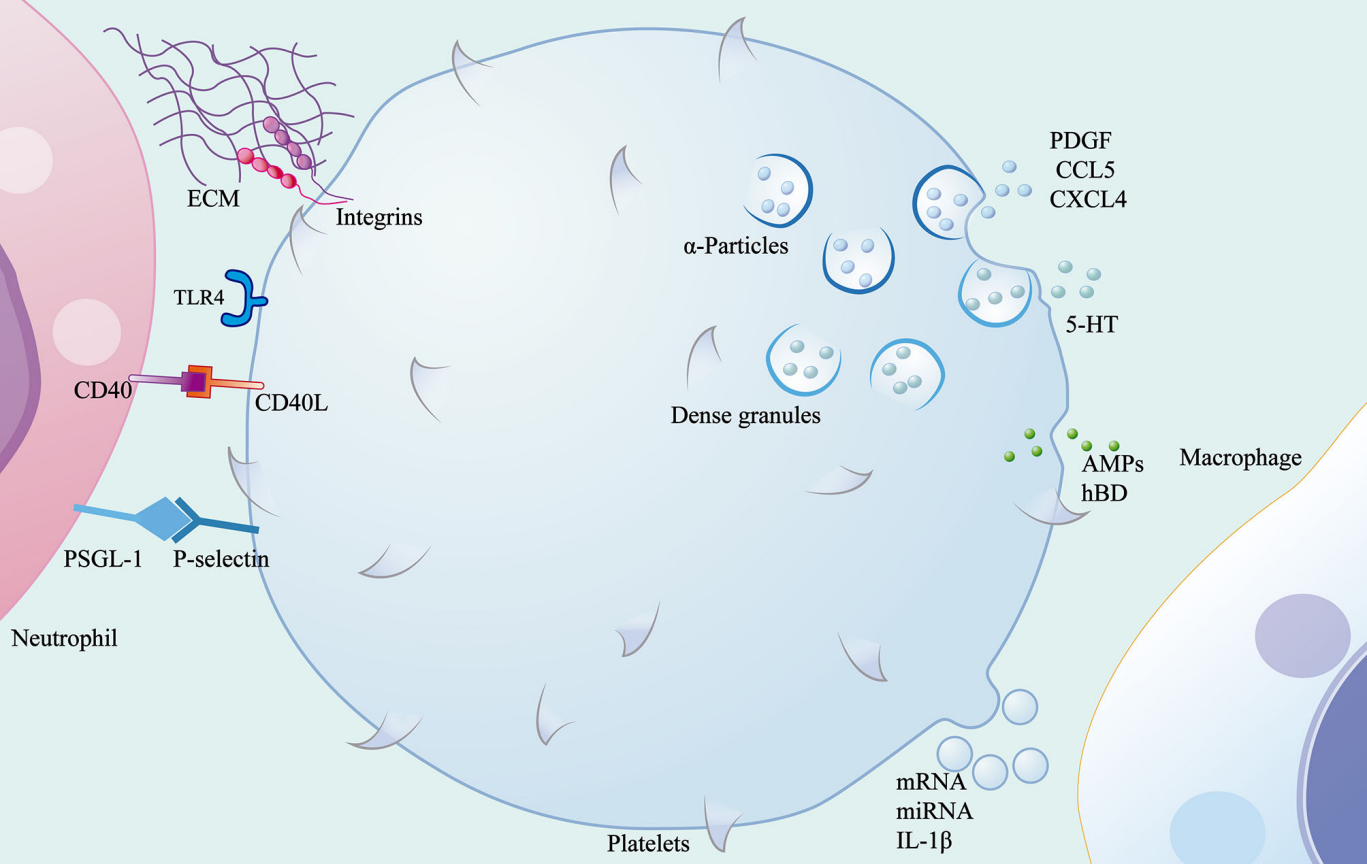
Role of the platelets in wound inflammation. Platelets mediate the migration of immune cells through contact with ECM and other cells through surface adhesion molecules and pattern recognition receptors, and release α-particles, dense granules, microvesicles and antimicrobial peptides.

In addition to playing a role in haemostasis and thrombosis, platelets are also involved in the regulation of immune responses during inflammation. Adhesion molecules and pattern-recognition receptors are distributed on the surface of these non-nucleated cells, enabling platelets to receive signals from the microenvironment and interact with other cells or the ECM. Activated platelets adhere to fibrinogen in the ECM *via* integrins β1, β2, and β3 on the cell surface. TLR4 and CD40L mediate the formation of NETs and the production of ROS (57, 58). Platelets have protein synthesis function that enables them to have bioactive protein libraries stored in their granules and vesicles and to release these stored proteins when activated by signals. A variety of chemokines and growth factors contained in α-particles can recruit immune cells from circulation, such as platelet-derived growth factor, platelet factor 4, chemokine C-C-motif ligand(CCL)5, and chemokine C-X-C-motif ligand(CXCL)4, which contribute to the recruitment and degranulation of neutrophils as well as the regulation of the polarization of macrophages (59). P-selectin, an adhesion molecule secreted by α- particles, interacts with P-selectin glycoprotein ligand-1 deficient(PSGL-1) expressed on immune cells as a key mediator of platelet-mediated immune cell rolling (57). The release of 5-HT from dense granules promotes the recruitment of neutrophils (59). In addition to the recruitment of immune cells, proteins containing defensins and antimicrobial peptides(AMPs) are released by platelets to contribute to the defence mounted against invading microbes (60). In addition, platelets release microvesicles after activation. These platelet-derived microvesicles not only deliver signalling molecules including mRNA and miRNA to the microenvironment, but also serve as the main pathway for the secretion of IL-1β by platelets (61, 62). Simply put, activated platelets release many proteins that help heal wounds. Platelets effectively promote wound healing by inducing the polarisation of macrophages from the pro-inflammatory phenotype to the repair phenotype (63). Interestingly, by exploring the protein heterogeneity within platelet particles, researchers have found that the protein uptake and packaging by platelets may be influenced by disease status (63). This may be the reason why studies have found that dermal matrix with platelets during wound inflammation can significantly promote fibroblasts proliferation and migration but has no obvious anti-inflammatory effect (64). The clinical application of platelet-rich plasma (PRP) in the field of regenerative medicine has been widely recognized and discussed. According to cell composition and fibrin content, platelet concentrators are divided into four categories: pure platelet-rich plasma (P-PRP), leukocyte-rich and platelet-rich plasma (L-PRP), pure platelet-rich fibrin (P-PRF) and leukocyte-and platelet-rich fibrin (L-PRF) (65). The results of a randomized controlled trial showed that L-PRP treatment after total hip arthroplasty could quickly close skin incisions (66). Huang et al. proposed a new type of platelet lyophilization therapy, which can improve wound healing rate and overcome the drawbacks of non-standardized and long-term PRP storage (67). To summarise, although researchers have realised that platelets are not only related to haemostasis, but also closely related to immune response, further studies are needed to confirm whether this association affects the local immune response or even wound healing efficiency during skin wound inflammation (**Figure 3**).

### Complement

The complement system is an intricate network of immune responses made up of a series of proteins that are activated after trauma to participate in the removal of microorganisms and damaged cells. In addition to the large amount of complement proteins provided by the liver, complement proteins are also produced in immune cells, fibroblasts and keratinocytes (68, 69). Complement proteins form C3 convertase through an alternative pathway, a lectin pathway or a classical pathway. C3 is then cut into small fragment C3a and large fragment C3b. C3b attaches to the pathogen surface in combination with C3 convertase to form C5 convertase, which then cuts C5 into small fragment C5a and large fragment C5b. Subsequently, C5b forms a membrane-attacking complex with C6, C7, C8, and C9, which produces holes in the surface of the pathogen, resulting in pathogen cleavage (70). Small fragments C3a and C5a act as effective inflammatory mediators to recruit inflammatory cells to the injured site (71). C3a also affects vasodilation and infiltration, resulting in local histamine release, vascular permeability and increased inflammatory cell infiltration; C5a has been shown to promote the migration and oxidative explosive power of macrophages and neutrophils during wound inflammation (72, 73). The application of C3 and C5 in collagen carrier alone or in combination was able to promote collagen deposition 3 days after trauma, thus increasing the maximum wound strength in a rat incision model, indicating the beneficial effect of complement (74–76). However, the increase of complement degradation factor C3d in burn wounds and C3 in the serum of patients with chronic leg ulcers suggested the negative effect of complement on wounds (77). In the study of a skin-wound model of C3 or C5 deficient mice, it was found that blocking the complement activation pathway could reduce the intensity of inflammation and accelerate the rate of early healing (78, 79). Similar results of rapid wound healing were observed in Cynomolgus monkeys treated with C3 inhibitor Cp40 (80). Excessive activated complement that affects wound healing in diabetic wounds can also be controlled by complement C1 peptide inhibitors to restore the level of wound healing (81). In addition, complement protein C1q was found to be involved in angiogenesis in wound healing independent of the complement activation pathway (69). Low-molecular-weight heparin tinzaparin sodium is used in the treatment of thrombosis-related diseases with its anticoagulating properties, but its potential to inhibit complement fixation and C5a liberation may improve skin inflammation (82). The above results suggest that the inhibition of complement activation is an effective way to stimulate wound healing. In clinical practice, although complement inhibitors have been used to treat patients with acute and chronic inflammation, no clinical studies have been done to test their actual efficacy in chronic wound of the skin (77).

## Role of Non-Inflammatory Cells in the Inflammatory Microenvironment

Non-inflammatory cells (keratinocytes, endothelial cells, and fibroblasts) in the wound microenvironment are mainly involved in neovascularisation, granulation tissue, and re-epithelialisation during proliferation and reconstruction (1). Thus far, their roles in these processes have been extensively reviewed. Interestingly, these cells are not idle in the inflammatory microenvironment, but there are few functional descriptions of their role in this phase. Keratinocytes and fibroblasts can secrete cytokines, chemokines, and bioactive material to participate in pathogen clearance and immune regulation. Furthermore, vascular endothelial cells use a well-known emigration mechanism to transport circulating immune cells to tissue.

### Keratinocytes and Fibroblasts

TLRs are transmembrane proteins that consist of leucine-rich repeats. Thirteen members of the TLR family have been identified and 10 of these are expressed in humans (6). As pattern recognition receptors, TLRs recognise pathogen-associated molecular patterns in response to microorganisms and damage-associated molecular patterns in response to tissue and cell damage. TLRs trigger pro-inflammatory responses, promote immune cell recruitment and antigen presentation and thus participate in local immune responses (83). They are expressed in most inflammatory cells of the skin, such as macrophages and DCs, and have been demonstrated in keratinocytes, fibroblasts, and other non-inflammatory cells (84). Epidermal keratinocytes have been shown to express TLR-1 through 6, TLR-9, and TLR-10, and fibroblasts express TLR-1 through 10 (6).

In intact skin, keratinocytes are closely connected to adjacent epithelial cells through desmosomes and to the extra cellular matrix of the underlying basement membrane through semi-desmosomes (85). After early skin injury, the TLRs of keratinocytes sense damage signals and secrete antimicrobial factors such as AMPs to clear pathogens (9, 86). In addition, keratinocytes activate the NF-kB pathway to release pre-stored cytokines such as IL-1α and TNF-α, during injury, initiating and amplifying the pro-inflammatory signal cascade (87, 88). Subsequently, keratinocyte-derived CCL27 and CCL20 allow recruitment of LC and T cells from circulation to the inflammatory site, while rapidly activating resident immune cells (88).

**Figure 4:**
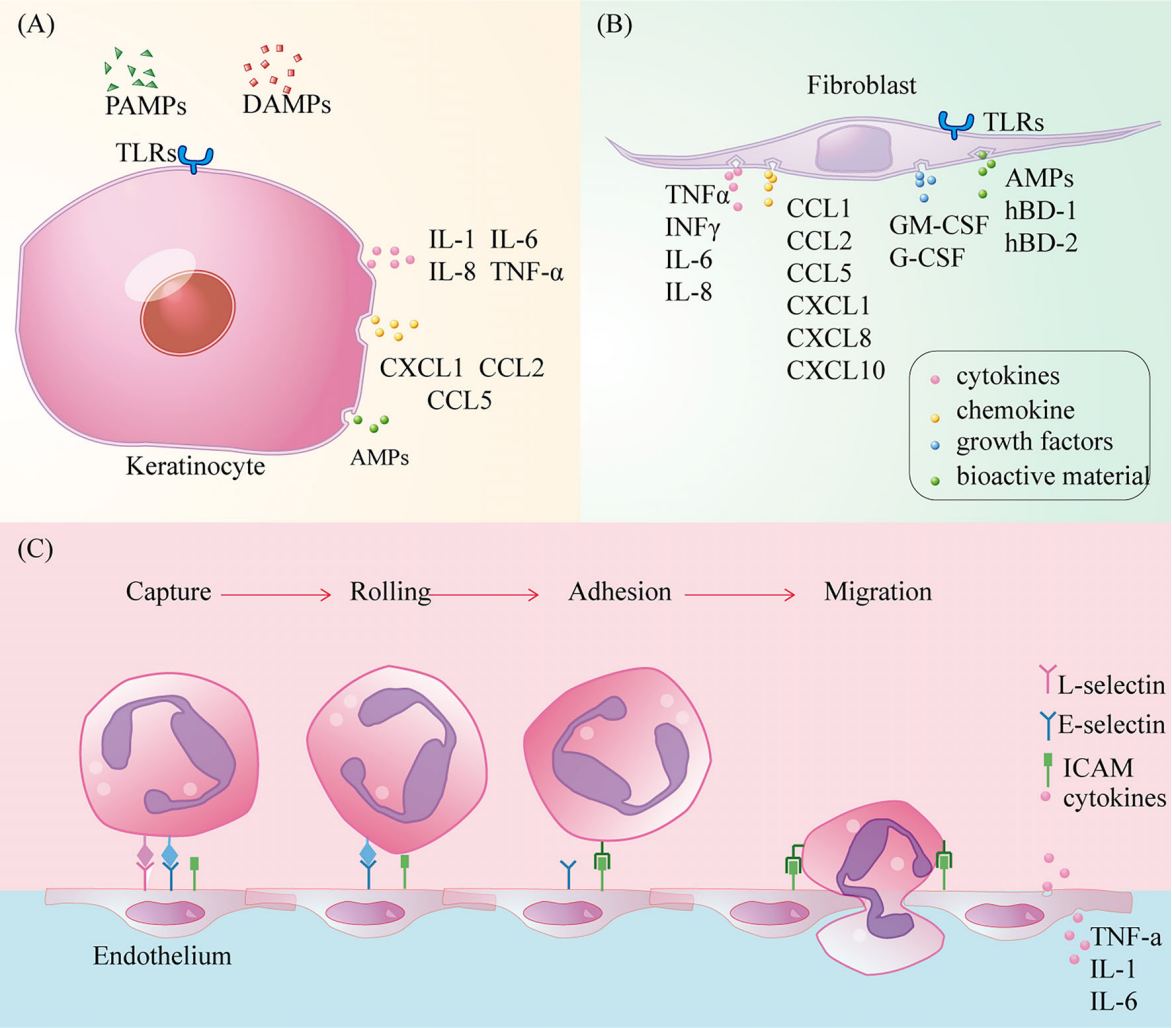
The role of keratinocytes, fibroblasts and vascular endothelium in wound inflammation. **(A)** Toll-like receptors activate cells to secrete cytokines, chemokines recruit immune cells, and synthesize antimicrobial peptides to kill pathogens; **(B)** Toll-like receptors activate cells to secrete cytokines, chemokines and colony-stimulating factors to recruit immune cells, while synthesizing antimicrobial peptides and defensins to kill pathogens, but also participate in new angiogenesis, conducive to immune cells clear and moist; **(C)** Circulating immune cells infiltrate into the injured tissue through capture, slow rolling, regulation of adhesion strength, crawling in the lumen, and eventually cross-cell or paracellular migration.

Fibroblasts, as dermal interstitial cells, repair wounds, mainly through cell proliferation and collagen deposition after tissue injury. The transcriptional analysis of fibroblasts has shown that many immune gene loci have transcriptional potential (89). Under the activation of IL-α released by keratinocytes and TLR ligands in the wound microenvironment, fibroblasts activate the NF-kB pathway for the induction and recruitment of inflammatory cells by proinflammatory cytokines (TNF α, INF γ, IL-6, IL-8), chemokines (CCL1, CCL2, CCL5, CXCL1, CXCL8, and CXCL10), and growth factors (granulocyte/macrophage colony stimulating factor (GM-CSF) and granulocyte colony stimulating factor (G-CSF) (4, 9, 90, 91). CCL2 is closely associated with monocyte recruitment (92). Like keratinocytes, fibroblasts also synthesise AMPs and defensins (hBD-1 and hBD-2) to clean wounds (9, 93). In addition, vascular endothelial growth factor-A, which is synthesised by fibroblasts, is involved in angiogenesis in the inflammatory microenvironment and can recruit monocytes/macrophages (94) (**Figures 4A, B**).

Although the immunomodulatory effects of the two abovementioned types of cells in the inflammatory period of wound healing can be affirmed, at present, skin grafts are mostly used to seal the wound by using their proliferation and differentiation abilities. Apligraf^®^ has been approved as an allograft in several countries for the treatment of acute wounds (95). Randomized controlled trials conducted by Poinas et al. have shown that biological dressings composed of foetal fibroblasts and keratinocytes show low immunogenicity and high regeneration efficiency, and they are expected to substitute skin allografts (96).

### Vascular Endothelial Cells

Vascular endothelial cells are a barrier and sensor between the circulation and basic tissue and are the key effectors that regulate the entry of circulating immune cells into the inflammatory microenvironment. Under the stimulation of wound injury signals, circulating immune cells can pass through vascular endothelial cells through the process of capture, slow-rolling, adhesion intensity regulation, intraluminal crawling, and finally cross-cell or paracellular migration, which is a dynamic balancing process (97, 98). Stimulated by pro-inflammatory factors, endothelial cells express L-selectin, E-selectin, and P-selectin, which bind to ligands (LFA-1 and Mac-1) on circulating immune cells (47). These mediate circulating immune cell capture from fast-flowing blood and slow them down as they adhere to endothelial cells. Subsequently, heterodimer adhesion molecules called integrins, such as intercellular cell adhesion molecule(ICAM)-1 and ICAM-2, mediate the strong adhesion of circulating immune cells to the vascular endothelium (98). Finally, circulating immune cells crawling on the surface of vascular endothelial cells infiltrate the wound microenvironment through the paracellular pathway under the influence of chemokines (99). Under normal conditions, the intact polysaccharide-protein complex/endothelial surface layer can be used to inhibit the adhesion of circulating immune cells to vascular endothelial surfaces (100). In addition, vascular endothelial cells produce many cytokines and chemokines, such as TNF-α, IL-1, and IL-6 (101). Although vascular endothelial cells expressing TLR-2, and TLR-4 can mediate inflammation through NF-κB and MAP kinases, the role of TLRs expressed by these cells in wound healing needs to be further elucidated (102) (**Figure 4C**). Among a large number of skin substitutes, vascular endothelial cells partially derived from adipose matrix can achieve vascular network regeneration in diabetic wounds, but there is still no study on the mechanism of their immunomodulatory effect (103).

## Role of the ECM in the Inflammatory Microenvironment

The ECM is a complex and dynamic structure that not only acts as a scaffold for cells and tissues but also interacts with cells to produce regulatory signals for cell migration, proliferation, differentiation, and apoptosis (104). ECM components play an important role in every stage of wound healing and are constantly remodelled during the repair process; however, this section focuses on the function of the ECM in the inflammatory microenvironment. In the case of tissue injury, the temporary matrix formed on the wound provides a scaffold for the infiltration of immune cells. Additionally, the ECM at the edge of the wound can transmit damage signals to immune cells and guide their infiltration. ECM scaffolds provide mechanical support for cell regeneration and tissue repair, creating a natural microenvironmental niche (105).

### Temporary ECM

In 1998, Magnusson et al. described the temporary ECM of wound healing as “early” stage and “late” stage (106). The early, temporary matrix is formed immediately after vascular injury and consists of platelets, plasma proteins [such as fibrin, fibrinogen, fibronectin (FN), hyaluronic acid (HA), heparin sulfate(HS)], which infiltrate the wound site, prevent blood loss, and provide a temporary scaffold for subsequent infiltration of immune and repair cells (106–109). Subsequently, neutrophils, macrophages, and lymphocytes successively enter the inflammatory microenvironment and establish the late temporary matrix (105, 106). Fibrin, which is derived from profibrinogen, is the main component of the early temporary matrix and is upregulated in response to injury and inflammation (110). In addition to providing the necessary cellular support for immune cells, fibrin can also bind to integrin receptors on the surface of immune cells to mediate cell migration, such as neutrophils and inflammatory macrophages that bind to fibrin through their αMβ2 integrin receptors (111). As repair progresses, more plasma FN is deposited and becomes the main component of the late temporary matrix (106, 110). A study of rat skin wounds showed that the level of FN mRNA increased significantly after injury (111). FN can stimulate cell migration and adhesion by connecting actin filaments through integrins (112). It can also activate macrophages to secrete TNF-α, IL-6, and IL-8 (87). Besides, proteoglycan HA and HS are also present in the temporary matrix. HA increases in early repair stages and can both activate inflammation as a proinflammatory factor and limit inflammatory damage. The former mainly mediates cell migration and increases inflammatory cell infiltration by binding to immune cell surface receptors (CD44, ICAM-1 and RHAMM), while the latter eliminates tissue damage caused by inflammation by scavenging free radicals and matrix degrading enzymes (113). The HA content in the foetal wound microenvironment is higher and lasts longer compared to adult wounds, and exogenous HA has a beneficial effect on wound healing (114). HS is a temporary substrate, produces growth factors needed during wound healing,such as fibroblast growth factor, platelet-derived growth factor and transforming growth factor-β (115). And it may play an important role in promoting the migration of neutrophils to the wound site (115).

**Figure 5:**
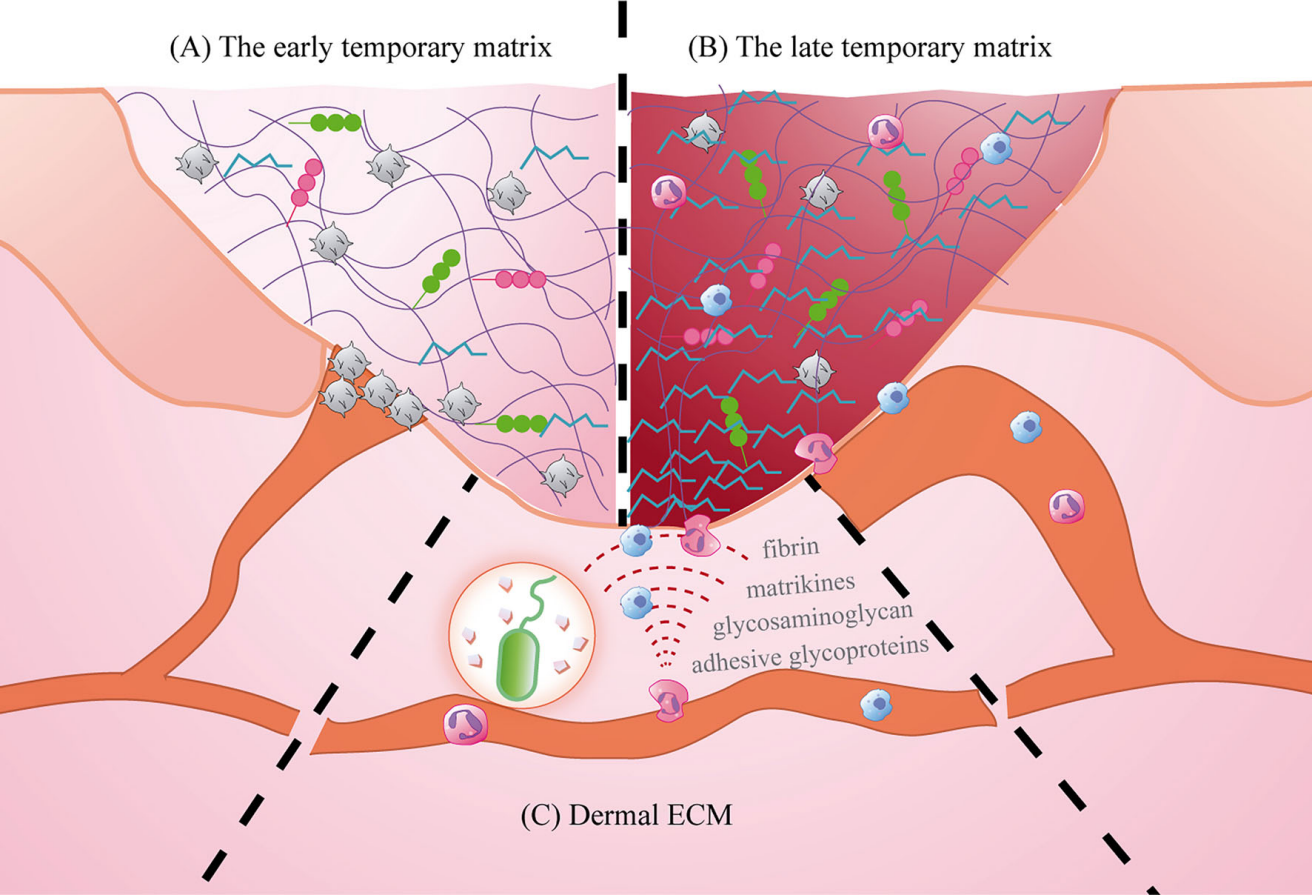
The role of ECM in the inflammatory microenvironment. **(A)** The early temporary matrix: mainly fibrin, playing the role of hemostasis and providing scaffold; **(B)** The late temporary matrix mainly plasma fibronectin, inducing cell activation and migration; **(C)** Dermal ECM: providing support and promoting cell migration.

After infiltrating the wound microenvironment through the vascular endothelium, circulating immune cells need to travel through the ECM to reach the edge of the wound and then infiltrate the temporary matrix. In this process, the ECM structure acts as a scaffold for immune cell adhesion and tissue structure, and produces cytokines, growth factors, and molecules and their derivatives, which promote the activation and migration of immune cells (116). Because epidermal cells are closely bound to the basement membrane, the ECM of the dermis is further explored here. Mechanical damage and pathogen invasion can cause the decomposition of macromolecular components such as fibrin, adhesive glycoproteins, and glycosaminoglycan in the ECM and release related molecules with pro-inflammatory functions (117). Matrikines are fragments produced by partial proteolysis, and these molecules and their derivatives in the ECM can coordinate the entry of immune cells into the inflammatory microenvironment by remodelling the tissue cytoskeleton and regulating signal transduction (118). At the same time, chemotactic gradients are formed on the wound surface to limit the access of specific cell subsets to the local inflammatory microenvironment (116) (**Figure 5**).

Based on the importance of the ECM in wound healing, biomaterials simulating ECM have been widely used in the clinic. ECM substitutes provide temporary support and migration sites for cells to close the wound. Harding et al. reported that a synthetic biomimetic acellular matrix was used as a substitute for ECM in the treatment of refractory leg ulcers, which proved its effectiveness and safety (119). In addition, ECM used to treat diabetic foot ulcers can produce clinical results similar to those of human fibroblast-derived dermal substitutes, but at a lower cost (120). It is worth noting that current research on a variety of biosynthetic materials is inseparable from the composition of the ECM, especially the application of fibrin and collagen.

### Mechanical Signalling of the ECM

Although the regulation of the behaviour and fate of immune cells by growth factors and signalling molecules in the ECM has been extensively studied, mechanical signalling is equally important in activating immune cells during tissue regeneration. The influence of the mechanical signals of the ECM on cell behaviour is mainly mediated by integrin receptors. Integrin is a bidirectional transmembrane receptor composed of α and β subunits (121). When mechanical signals from the ECM generated by tissue injury induce conformational changes in the extracellular domain of integrins, activated integrins activate intracellular signalling pathways to regulate gene expression (122, 123). Activated integrins recruit a large number of mechanically sensitive proteins (such as Talin and Vinculin) to assemble into nascent adhesions (NAs) and deliver a reverse thrust to the ECM *via* actin. Subsequently, NAs degrade rapidly, and adherent plaque kinase recruits ARp2/3 (actin cytoskeletal nucleating agent) to form adherent plaque (FAs) to drive cell migration (124). αDβ2 and αMβ2 are expressed on the cell surface of macrophages and can migrate to inflammatory sites in response to the mechanical stress of the ECM (125, 126). In addition, activation of mechanically gated ion channels has been shown to play a role in mechanical transduction of immune cells, for example, Piezo1 and instantaneous receptor potential cation channels (TRP). Piezo1 channels on macrophages, activated by mechanical stimuli, mediate the influx of Ca^2+^, resulting in the production of pro-inflammatory mediators such as IL-6, TNF-α and prostacyclin E2 (123). Research shows that Piezo1-deficient macrophages reduce inflammatory cell infiltration during wound healing (127). TRPV4 is the most studied mechanically gated TRP channel and is widely expressed in immune cells. Activated TRPV4 binds to F-actin to mediate the extravasation of neutrophils and macrophages into injured and inflammatory sites (128). In response to mechanical signals from the ECM, cytoskeletal F-actin mediates the entry of cardiac-associated transcription factors (MRTF) and YAP/TAZ transcription factor complexes into the nucleus to regulate gene expression. Nuclear expression of YAP and the NF-κB pathway crosstalk up-regulate the expression of pro-inflammatory cytokines in macrophages (129). Therefore, the proliferation and migration of immune cells require the generation and transmission of mechanical forces. The resting stress on human skin triples during wound healing (130). However, recent studies have found that the rigidity of the ECM environment is more likely to lead to excessive inflammation and scar hyperplasia (127). This idea was supported by skin damage studies in Prickly African mice (Acomys), where in high levels of repair were associated with low levels of inflammation associated with resting hypotonia (130). However, some researchers have proposed that the existing models for simulating ECM structural remodeling do not take into account the mechanical plasticity caused by the change of viscoelasticity of ECM with time. They proposed a discrete model to study the effects of ECM viscoelasticity on matrix remodeling and stress distribution (131). Therefore, in order to simulate the role of physiological ECM in wound healing, the mechanical interaction between cells and ECM should be considered in the synthesis of ECM biomaterials.

### Physicochemical Factors of the ECM

Although the efficacy of physical therapy has widely recognised in the clinical treatment of wounds, the regulatory mechanism of this type of therapy has not yet been clearly elucidated. In addition to mechanical forces, other physicochemical factors of the ECM (humidity, temperature, oxygen, and pH) also regulate immune responses. Winter et al. proposed the concept of treating wounds with a moist microenvironment in 1962 (132). This is essentially the same as the traditional surgical therapy of “oasting pus to promote regeneration” in traditional Chinese medicine, which emphasises that wound healing requires a wet microenvironment. Maintaining a wet microenvironment for the wound is beneficial to the migration of inflammatory cells (133, 134). The moist environment created by saliva has been associated with rapid oral wound healing (135). Therefore, after determining and adequately treating the cause of the wound, maintaining moisture in the wound area can better promote healing. The use of a combination of collagen and foam helps to maintain the moist environment of the wound in patients with venous ulcers of the lower extremities, increase the perfusion of blood and oxygen into the wound, and thus promote angiogenesis (136). The temperature of normally healed wounds peaked 3 days after injury and then gradually decreased (137, 138). The wound temperature increases due to local dilation of blood vessels, allowing the body to deliver more oxygen and nutrients to the injured area (139). Low temperature may weaken the oxidative killing ability of neutrophils and the motility of macrophages, which may easily lead to wound infection (140, 141). Infrared light irradiation is a traditional wound care method, its principle is that it can promote blood circulation to accelerate wound healing through heating. Placing diabetic ulcer patients in a heated warm room can significantly improve the wound healing rate (142). Adequate oxygen is required for phagocytes to produce antibacterial reactive oxygen, so adequate oxygen is crucial for immune cells to phagocytose invading microorganisms (139, 143). The purpose of oxygen therapy is to provide an oxygen-rich microenvironment for wound healing, such as hyperbaric oxygen therapy, which is widely used in clinic. However, due to its limitations, researchers are developing a variety of oxygen-producing biomaterials as a source of oxygen release (144). Acidic pH enhances acute inflammatory responses by stimulating the migration and aggregation of neutrophils and macrophages, delaying spontaneous apoptosis, and prolonging the functional life of neutrophils (145, 146). In contrast, alkaline pH has been shown to adversely affect wound healing by reducing oxygen in the wound and providing an environment conducive to bacterial growth as well as by significantly inhibiting neutrophil motility (138, 147). Honey has been very popular in wound treatment since ancient times. The low PH value of honey can increase the oxygenation of the injured site, remove free radicals and promote healing (148).

## Cell-Cell and Cell-ECM Interactions

**Figure 6:**
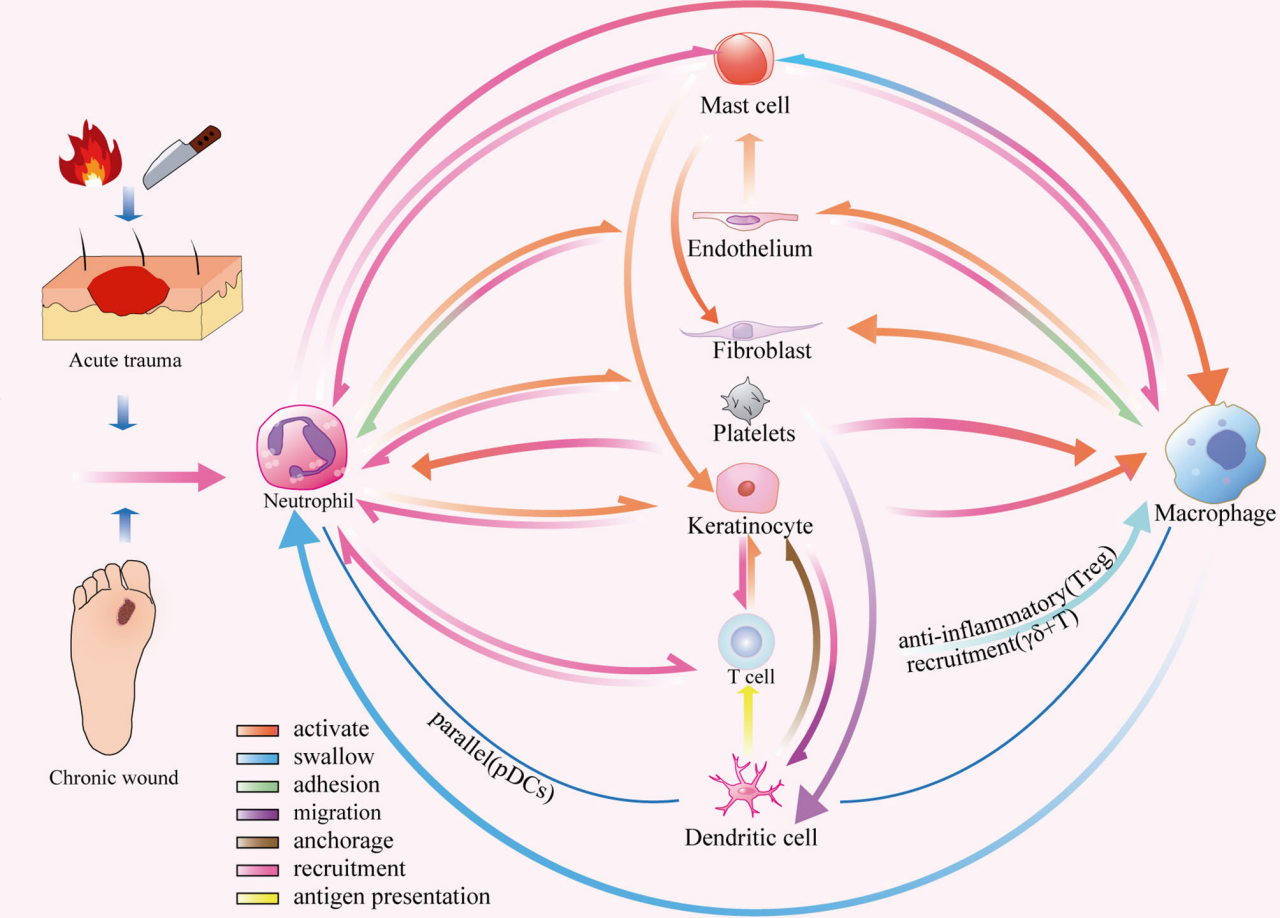
Dynamic network of cell-cell interactions in an inflammatory microenvironment.

After tissue injury, the platelet cascade reaction activates hemostasis and the complement system, which eventually leads to wound healing in the inflammatory phase. When circulating immune cells migrate to the inflammatory microenvironment, they must first adhere to vascular endothelial cells and bind to selectins, integrins, or adhesion molecules (30, 99). This binding is an important condition for the release of TNF-α-mediated neutrophils, which increase vascular permeability (98). They also secrete factors such as thrombin, which promote mast cell migration, proliferation, and local differentiation (30). Neutrophils in the early inflammatory microenvironment recognise damage signals and generate H2O2 gradients through respiratory bursts that initiate an inflammatory cascade. This process occurs in combination with cytokines and chemokines (e.g. CXCR1, CXCR2, and CCR1) that are released by activated macrophages, epidermal γδ+T cells, endothelial cells, fibroblasts, and keratinocytes (10, 14, 16, 50, 51). Rapid and transient accumulation of PDCs recruited by damaged keratinocytes occurs in parallel with increased neutrophil infiltration (49). Subsequently, mast cells are recruited and release trypsin-like enzymes, which bind to the protease-activated receptor 2 on endothelial cells, causing vasodilation and further amplification of the inflammatory response (5). This series of reactions promotes the accumulation of M1 macrophages in the injured site and plays a role in the phagocytosis of apoptotic cells and pathogens. Meanwhile, LCs anchored to adjacent keratinocytes by the adhesion molecule E-cadherin migrate from the epidermis to the dermis in response to CXCL12 from dermal fibroblasts and MCP-1 from keratinocytes (40, 44, 149, 150). They provide antigens to the immune response of memory T cells, which are recruited by CCL27 and produced by activated keratinocytes in the skin (151). However, a dynamic balance in the inflammatory response is necessary for active wound healing (55). Tregs can restrict the aggregation of pro-inflammatory macrophages, and M1 macrophages engulf apoptotic immune cells, indicating a transition from the inflammatory to the proliferative microenvironment of the wound surface (**Figure 6**).

The ECM constitutes a place for tissue repair following trauma, and its macromolecules and their derivatives can regulate the activation and migration of inflammatory cells as well as the proliferation and differentiation of repair cells. The ECM component HS participates in immune cell migration and regulates the secretion of cytokines, chemokines, and growth factors (152). HA is involved in the regulation of macrophage phenotype (153). Fibrin and FN mediate monocyte migration and secretion of TNF-α, IL-1β, and IL-6 and stimulate the migration and adhesion of fibroblasts, keratinocytes, and endothelial cells through integrin receptors (104, 116). Various factors and enzymes released by activated cells can constantly supplement and reshape the components of the ECM (116, 154). Matrix metalloproteinases and other enzymes released by activated inflammatory cells can expose the migration sites of the ECM by cleavage of the basement membrane (105). The release of toxic particles by neutrophils causes the rapid degradation of fibrin by plasmin and elastase, and the degradation products can induce or amplify the inflammatory process (154). The factors and proteins expressed by repair cells can fill the defects in the ECM and supplement the cell factor library of the ECM. The ECM and its derivatives help to regulate the biological function of cells in the microenvironment, while factors, enzymes, and proteins secreted by cells modify the components of the ECM to form a dynamically balanced inflammatory microenvironment for wound healing. There are many kinds of ECM biomimetic materials for simulating wound repair that are rapidly being developed. The incorporation of fibrinogen and collagen I into biomimetic coaxial nano-scaffolds to solve wound inflammation significantly promoted wound repair (155). Taking advantage of the ECM scaffold potential, the ECM modified with silver nanoparticles (nAg) has both antibacterial and tissue regeneration abilities (156). However, the ECM is constantly being supplemented and reshaped, and the dynamic adjustment of the composition of the ECM to simulate the microenvironment needs further research and exploration.

Active wound healing depends on an orderly and moderate immune response regulated by inflammatory cells, non-inflammatory cells, and the ECM. Disruption of any of these links can lead to persistent inflammation and prevent the repair process, leading to the occurrence of chronic wounds such as chronic venous ulcers, diabetic foot ulcers, and pressure sores. These chronic wounds occur in the context of venous hypertension, diabetes, or angiopathy caused by prolonged tissue pressure (157). The destruction of vascular endothelial cells reduces the amount of blood perfusion to the wound area and causes tissue hypoxia (158). At the same time, the number of platelet-derived microvesicles in peripheral blood increases, and these microvesicles are internalised by vascular endothelial cells, thus up-regulating the expression of intercellular adhesion molecule-1 and resulting in more immune cells extravasating to the injured site (159). Neutrophil recruitment promotes the production of more NETs and activates the release of NLRP3 inflammatory bodies and IL-1β of macrophages through the TLR-4/TLR-9/NF-κB signal pathway (160). Although a large number of pro-inflammatory M1 macrophages are activated, phagocytosis of apoptotic neutrophils by these macrophages is inhibited, resulting in a disturbance in the process of macrophage polarization (161–163). With increase in the number of degranulated mast cells, a sustained inflammatory response is eventually achieved (164). The inflammatory cascade continues to cause the activation and accumulation of T cells, especially that of Th17 cells. Th17 cells secrete IL-17 to maintain the activity of M1 macrophages, leading to persistent inflammatory wound healing (162). On the contrary, increasing the number of LCs in the wound of diabetic mice has been shown to improve healing (165). A large number of pro-inflammatory factors increased the release of MMPs and accelerated the degradation of the ECM, which further reduced the proliferation and collagen deposition of fibroblasts (4, 166). In this pathological inflammatory microenvironment, inflammatory genes expressed by fibroblasts are up-regulated and keratinocytes release IL-1α and type I interferon, triggering an inflammatory chain reaction in adjacent stromal cells (92, 162). This, in turn, promotes the flow of immune cells into the injured site. In summary, the delay of chronic wound healing can be attributed to the failure of ECM remodelling caused by the uncontrolled immune regulation of inflammatory and non-inflammatory cells.

## Discussion

We briefly reviewed the effects of the interaction of inflammatory cells, non-inflammatory cells, and the ECM on the inflammatory microenvironment of skin wounds. The spatial coexistence of functionally specialised cell types is a basic feature of multicellular organisms, and the different anatomical locations and degrees of interaction with other cells determine the functional differences in similar cells during the process of wound healing (i.e. cellular heterogeneity). For example, before circulating macrophages arrive, some tissue resident macrophages are already involved in the inflammatory response (16). Although different fibroblast subsets in human skin have been revealed, the unique gene expression changes in this population in skin wounds, especially in the inflammatory microenvironment, remain unknown (167). In addition, a wide range of cell types in wounds have significant plasticity under the influence of the ECM, which complicates the dynamic study of different cell subsets and their effects on wound healing. Single cell technology and space transcriptome technology, which are widely used because of the development of genomics technology, can solve the above problems step by step. The development and application of multiple sequencing technology can realise the unsupervised analysis of interactions among different cell types in skin wounds on the basis of restoring the spatial characteristics of tissue structure *in vivo (* 168).

Correct and coordinated actions in the inflammatory microenvironment ensure proper wound healing. However, when this process is disrupted, skin healing is delayed, which eventually leads to chronic wounds. Compared with acute wounds, marrow-like cells (neutrophils and macrophages) in chronic wounds exist in large numbers for a long time, accompanied by the degranulation of mast cells and high expression of inflammatory T cell subtypes (169). The signal molecules and proteases secreted by these immune cells can also lead to the over-expression of pro-inflammatory cytokines and chemokines and continuous degradation of the ECM in non-inflammatory cells such as keratinocytes (170). Although many research groups have successfully promoted healing by regulating inflammatory cells, it is possible to induce an inappropriate immune response by directly regulating the immune system. Therefore, designing functional regulation based on non-inflammatory cells and the ECM provide a potential therapeutic approach for chronic wound healing.

Understanding the inflammatory microenvironment of skin wounds will help to develop a comprehensive understanding of the processes affecting wound healing. This information can provide personalised guidance for the strict control of wound immune response. In particular, research on the immune function of non-inflammatory cells and the ECM in wounds may reveal new strategies for the clinical development of innovative wound treatments.

## Outstanding Questions

Several studies have shown that the inflammatory microenvironment is composed of inflammatory cells, non-inflammatory cells, and the ECM, which collaborate to scavenge necrotic cell fragments and prevent infection. The activation of repair-related cells promotes wound healing through the secretion of cytokines that lead to the proliferation stage. However, there is a need for further evidence to support the interactions between non-inflammatory cells and the ECM, which are involved in the immune regulation of the inflammatory microenvironment. Additionally, the functional advantages of all the inflammatory factors that control the inflammatory response needs to be elucidated. As the evidence that the reduction of inflammation can promote wound healing comes from the study of inflammatory cells, it is not clear whether the regulation of non-inflammatory cells and ECM can achieve the same or perhaps even a better immunomodulatory effect.

## Author Contributions

Literature search and manuscript drafting, ZW and GX. Figures, ZW and HL. Manuscript editing and revision, GX, FQ, and DW. All authors contributed to the article and approved the submitted version.

## Funding

This work was supported by the National Natural Science Foundation of China [grant numbers 81871570 and 82072195], the Science and Technology Plan Project of Guizhou Province [grant numbers (2020)4Y148], the PhD Fund of Scientific Research Foundation of Affiliated Hospital of Zunyi Medical University[grant numbers (2020-03)], and the Chinese Ministry of Education [grant numbers 2020-39].

## Conflict of Interest

The authors declare that the research was conducted in the absence of any commercial or financial relationships that could be construed as a potential conflict of interest.

## Publisher’s Note

All claims expressed in this article are solely those of the authors and do not necessarily represent those of their affiliated organizations, or those of the publisher, the editors and the reviewers. Any product that may be evaluated in this article, or claim that may be made by its manufacturer, is not guaranteed or endorsed by the publisher.
